# Of Fever from Venereal Poison

**Published:** 1800-03

**Authors:** J. Haygarth

**Affiliations:** London and Edinburgh


### *Of Fever from Venereal Poison; by* J. HAYGARTH, M. D. F. R. S. London and Edinburgh.

### *To the Editors of the Medical and Phyſical Journal.*

GENTLEMEN,

IN three ſucceſſive inſtances, I have obſerved, that the venereal poiſon applied to a finger, has produced a *low nervous fever*. How far ſuch an effect of this manner of infection is noticed by authors, I have not leiſure to inquire; it is, certainly, not generally known nor ſuſpected. In all the following caſes, my patients were Surgeons and Accoucheurs in extenſive practice.

#### THE FIRST CASE.

I was deſired to viſit a Medical Friend on the 25th of September, 1791. For a month, he had obſerved a browniſh, moveable tumour, of the ſize and ſhape of a horſe bean, on the back of the index of the right hand. It had remained in the ſame ſtate for many days; then it gradually became more painful. Ten days before I ſaw him, it had broken, and diſcharged a ſerous fluid. For five days paſt, he had obſerved a painful tumour in the right arm-pit, with a red, painful, and ſwelled line, which could be traced up the arm to the axilla, in the courſe of the lymphatic abſorbents. At times, he felt a violent pain darting from the ulcer of the finger, up the arm, to to the axillary gland. At the end of a month from the firſt appearance of the ſwelling on the finger, it became ulcerated, without any previous regular ſuppuration, and the ſore had an ugly, ſpungy aſpect, with an irritable and extremely painful ſurface.

For three days previous to my firſt viſit, he had felt the ſymptoms of a low nervous fever; as, alternate chilly and burning fits; ſhort but profuſe ſweats; laſſitude; extreme depreſſion of ſpirits; and a ſlight delirium.

On firſt viewing the ulcer, I was ſtruck with the idea that it was venereal. But fever, which appeared to be connected with the ulcer, was thought to be ſo unuſual a connexion, as conſiderably to diminiſh the probability of ſuch a cauſe. The fever alſo formed a ſtrong objection to the uſe of mercury. My patient had an excellent underſtanding, and had been long engaged in extenſive practice. He very judiciouſly urged theſe points with me. Among other arguments, he obſerved that the ſkin where the tumor and ſubſequent ulcer had formed, had previouſly ſuffered no injury, but was perfectly ſound.

After diſcuſſing this point with my patient for about five days, during which the ulcer increaſed in ſize and ſoreneſs, I obtained his conſent to uſe mercury. But, juſt at this time (on the 30th of September) he had a violent ſhuddering fit, with an increaſe of his other febrile ſymptoms. A general efloreſcence immediately appeared, and more particularly on the face, which had manifeſtly a venereal aſpect.

Such a formidable acceſſion of fever, at this critical time, induced me to ſuſpend the uſe of mercury till the 4th of October. During this interval of four days, the venereal blotches appeared, which clearly confirmed my former opinion of the nature of the poiſon. The ulcer rapidly advanced ſo as to endanger the loſs of two joints of the finger. To preſerve them, and the life of my friend, which at that time were both in the moſt perilous ſituation, the mercurial ointment was employed in larger quantity than, I believe, I ever directed in any other caſe. Salivation was excited. The ulcer healed in October, but violent ſhooting pains remained in the hand and arm.

At this time he was induced to attend ſome preſſing engagements in his profeſſion, though greatly fatigued with the leaſt exertion, and many of the venereal ſores, particularly on the face, were not healed. After going about for ſome weeks, he was ſeized with ſuch a violent pain in his legs and knees that he could never obtain ſleep for fifteen minutes together. He had the firſt night of good ſleep in the middle of April, after a long courſe of mercury, and the compound decoction of ſarſaparilla.

It

It is hardly neceſſary to obſerve, that this long protracted diſeaſe plainly proceeded from the urgent profeſſional duties in town and country, by day and night, which my patient was anxious to fulfil, ſo as to interrupt the ſeaſonable and regular uſe of proper remedies.

### The Second Case.

I was conſulted by another eminent Surgeon and Accoucheur, on the 9th of May, 1792. On the point of the fore finger of his right hand there had been a ſlight wound. For eighteen days it had gradually become a ſpongy and painful ulcer. In the axilla he had perceived a tumuor which gave him conſiderable pain. He had chilly fits, with a ſenſe of much debility, being ſcarcely able to walk to my houſe.

On this occaſion I aſſured my patient that his diſorder proceeded from venereal infection. My opinion was deciſive; not only from the appearance of the ulcer, but was much ſtrengthened and confirmed by my experience in the former caſe. He argued, and judiciouſly, from many ſymptoms which he enumerated, that he had a typhus fever; he aſcribed it to ſome putrid abſceſſes which he had lately opened. In conſequence of this idea, he thought that bark and wine were his proper remedies. With opinions totally oppoſite to each other, we ſeparated; but he ſoon returned to me, and conſented to begin a mercurial courſe, with a decoction of ſarſaparilla. I have reported in my caſe book, on May 27th, that he had uſed the remedies, but not regularly; that the wound of his finger was nearly healed, and the axillary tumour almoſt gone. The pain had ceaſed. He was deſired to continue the remedies; and his health was ſoon reſtored.

Some time after our firſt conſultation on the caſe, my patient acquainted me, that a woman whom he had lately delivered, had applied to him to cure her of ſome venereal ulcers. He entertained not a doubt that he received the venereal infection from theſe ulcers.

### The Third Case.

On the 19th of May, 1794, I was conſulted by another Accoucheur. I have noted, that a wound of a finger of his left hand had received a venereal infection three weeks ago. He complained of laſſitude, proſtration of ſtrength, giddineſs, and ſenſibility to cold. For a fortnight, he had perceived a tumour in a lymphatic gland above the elbow, and in another near the elbow. I adviſed Ung. Hydr. ʒß every night, with decoct. ſarſap.

April 8th, he had uſed eight ſcruples of mercurial ointment. His

His health had improved. The lymphatic glands, lately fwell-ed and painful, were relieved.

April 19th, he had ufed an ounce of the mercurial ointment. He was recovering; but the ulcer of his finger ftill remained red and fwelled. He was defired to continue the remedies.

Thus, in three fucceffive cafes, the venereal poifon applied to a finger, produced fordid, fpongy, painful, phagedenic ulcers; inflamed lymphatic abforbents and glands; and manifeft fymptoms of a low nervous fever.

Accoucheurs ought to be fully aware of the danger to which their health and life are fo much expofed. I have very feldom witneffed an inftance where the life of a patient was refcued from more imminent danger than in the firft cafe above related. Since thefe events have occurred to my obfervation, a painful doubt has frequently arifen in my mind, whether the death of my much-lamented friend, Hewson, might not be afcribed to fuch a fatal miftake.

I requeft the favour of you, Gentlemen, to infert thefe facts in your ufeful Mifcellany; and wifh to invite fome of your numerous and refpectable readers and correfpondents to relate, in your Journal, fimilar cafes which may have fallen under their obfervation, fo as to form fome general eftimate, whether fuch a fever frequently, or but feldom, accompanies the introduction of this poifon into the fyftem, when applied to a finger, lip, &c.* It may be prefumed, that fever is not the ufual confequence of this mode of receiving the venereal infection; otherwife, it muft have been more generally known among medical men. At any rate, the preceding cafes will give an ufeful warning to Surgeons and Accoucheurs; they will, on a like occafion, be aware of the danger, and be prepared with the proper remedy. If thefe hints preferve my profeffional brethren from miftakes which might prove fatal to themfelves, I fhall be gratified with the moft cordial fatisfaction; and am,

Gentlemen,

Your faithful fervant,

*Bath, Feb.* 7, 1800. J. HAYGARTH.

---

* On the 16th of October, 1769, I attended a young lady, whofe cafe I have noted: "For five weeks fhe has had a callous ulcer on her upper lip, probably venereal, with red and rough eruptions on her face and body. Pulfe 100; chilly on expofure to cold. She feels a fenfe of fatigue, but yet can walk and ride."

